# Antenna-Specific Glutathione *S*-Transferase in Male Silkmoth *Bombyx mori*

**DOI:** 10.3390/ijms15057429

**Published:** 2014-04-29

**Authors:** Xiang Tan, Xiao-Ming Hu, Xiao-Wu Zhong, Quan-Mei Chen, Qing-You Xia, Ping Zhao

**Affiliations:** 1 State Key Laboratory of Silkworm Genome Biology, Southwest University, Chongqing 400716, China; E-Mails: xiangtan@swu.edu.cn (X.T.); xiaoximingpan@126.com (X.-M.H.); chenquan@swu.edu.cn (Q.-M.C.); xiaqy@swu.edu.cn (Q.-Y.X.); 2 Medicine Research Center, North Sichuan Medical College, Nanchong 637000, Sichuan, China; E-Mail: zxw_strive@163.com

**Keywords:** glutathione *S*-transferase, antenna-specific, male silkmoth, detoxification, *Bombyx mori*

## Abstract

Glutathione *S*-transferases (GSTs) are multifunctional enzymes that are widely distributed in different species. GSTs detoxify exogenous and endogenous substances by conjugation to reduced glutathione. We characterized BmGSTD4, an antenna-specific GST, in male silkmoths. The full-length mRNA of *Bmgstd*4 was cloned by RACE-PCR and contained an open reading frame of 738 bp encoding a 245 amino acid protein. The antenna specificity of BmGSTD4 was validated at the mRNA and protein levels and BmGSTD4 was shown to localize in the sensillum of male silkmoth antennae. Homology modeling and multi-sequence alignment suggested that BmGSTD4 was a typical GST belonging to the δ class and had a canonical GST fold with a conserved *N*-terminus, including a glutathione-binding site and a *C*-terminal domain harboring a hydrophobic substrate-binding site. Restricted expression of BmGSTD4 in silkmoth antennae combined with GST activity suggested that BmGSTD4 was involved in the detoxification of harmful chemicals.

## 1. Introduction

Glutathione *S*-transferases (GSTs; *E.C.*2.5.1.18) are conserved enzymes that are widespread in both prokaryotes and eukaryotes. GSTs are phase II enzymes in the detoxification and metabolism of toxic compounds [1–3] that catalyze xenobiotic and endobiotic compound detoxification by conjugation with reduced glutathione (GSH). This process results in water soluble, less toxic conjugates that can be excreted. GSTs also have peroxidase [4] and isomerase activity [5], and are involved in regulating cell signaling pathways and biosynthesis [1]. Some GSTs noncatalytically bind endogenous and exogenous ligands [6–8].

The three major GSTs are cytosolic GSTs, microsomal GSTs (also called membrane-associated proteins in eicosanoid and glutathione metabolism; MAPEG), and mitochondrial GSTs (also known as kappa class GSTs). Based on the nomenclature of the mammalian cytosolic GSTs [9], insect cytosolic GSTs are subdivided into six classes: δ, ɛ, ω, θ, σ, ζ, with some GSTs remaining unclassified [10]. The δ and ɛ classes are insect specific. Insect GSTs have been studied for insecticide resistance, especially for artificial insecticides, such as dichlorodiphenyltrichloroethane and pyrethroid [11].

Previous studies identified and characterized GSTs related to olfaction or GSTs that are olfactory specific. These GSTs protect the olfactory system, but can have other functions. These kinds of GSTs were first found in the olfactory epithelium of cattle [12] and rats [13]. Rat GSTs are proposed to mediate the covalent modification of odorants for neutralization and clearance [14]. The olfactory-specific GST msolf1 in *Manduca sexta* protects the olfactory system from harmful xenobiotics and inactivates odorants [15]. A cDNA fragment for *Hagst-olf*, an antenna-specific gene in *Helicoverpa armigera*, was recently cloned [16]. A specific GST was shown to be preferentially expressed in the chemosensory organs of the swallowtail butterfly, *Papilio xuthus* L. [17]. In addition, olfactory GSTs from coho salmon (*Oncorhynchus kisutch*) have been cloned, expressed, and analyzed [18].

We identified BmGSTD4, a GST specifically expressed in antennae of male silkmoth, *Bombyx mori*. We cloned the full-length mRNA of *BmGSTd*4, expressed and purified recombinant BmGSTD4. This antenna-specific GST had GST activity, suggesting its importance for protecting antennae from harmful xenobiotics.

## 2. Results

### 2.1. Cloning and Analysis of Bmgstd4

**Figure 1.**
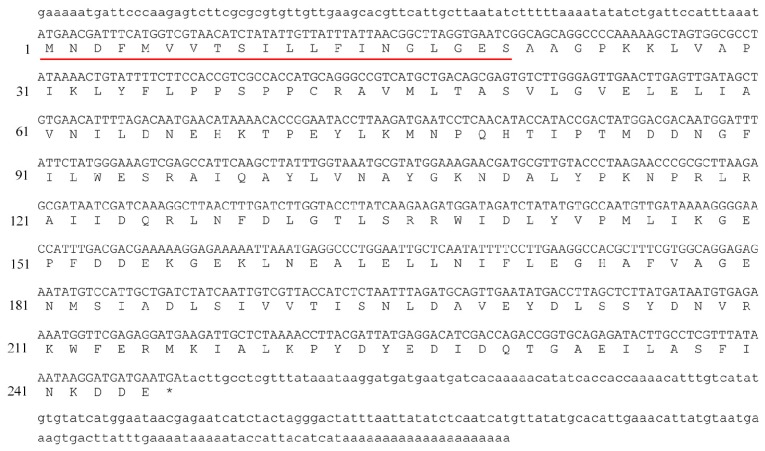
Nucleotide and deduced amino acid sequences of BmGSTD4. Full length *Bmgstd*4 mRNA and deduced amino acids. The 5′- and 3′-lowercase characters show the 5′- and 3′-UTR. Red underline: signal peptide.

*Bmgstd*4 was cloned from total male silkmoth antenna RNA by 5′- and 3′-RACE-PCR. *Bmgstd*4 mRNA was 1049 bp (Figure 1) long and contained a 738 bp open reading frame (ORF) with a 90 bp 5′-UTR and 221 bp 3′-UTR, a tail signal (AATAAA), and a poly(A) tail. The putative *Bmgstd*4 gene encoded a 245 amino acid protein with an *N*-terminal signal peptide of 22 amino acids. These features made BmGSTD4 different from cytosolic GSTs isoforms identified from silkworms. After removal of the signal peptide, the calculated molecular weight of the mature polypeptide was 25,518 Da and the theoretical pI was 4.72. BLAST of the putative conserved domains showed that BmGSTD4 was a typical GST in the δ class with an *N*-terminal GSH binding site and a *C*-terminal hydrophobic substrate-binding site.

### 2.2. Phylogenetic Analysis of BmGSTD4 with Other Insect GSTs

**Figure 2.**
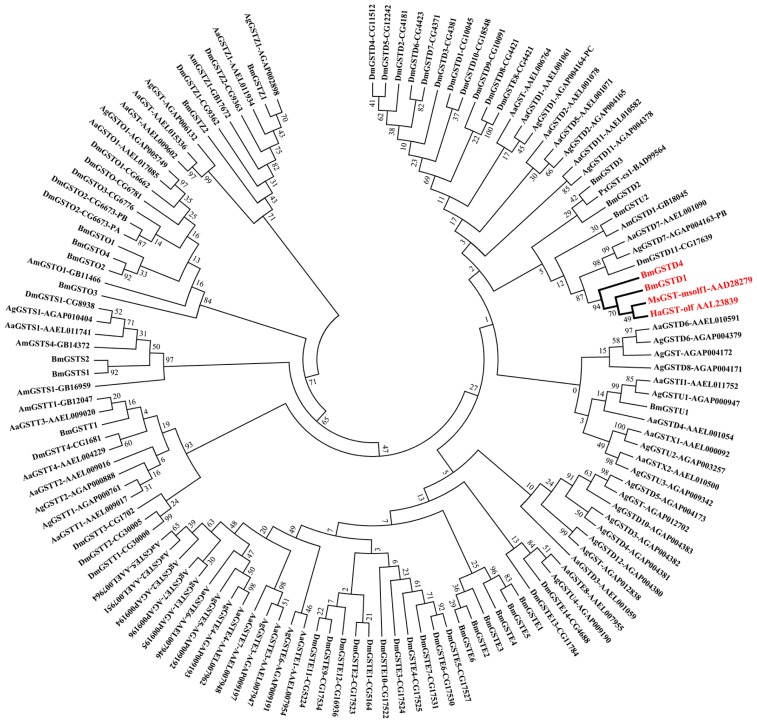
Phylogenetic tree of insect GSTs. Alignment of 127 amino acid sequences was used to construct the phylogenetic tree. Bold red, cluster formed by BmGSTD1, BmGSTD4, MsGST-mslof1, and HaGST-olf. Characters after protein denotation, accession numbers in NCBI. *B. mori* (Bm), *D. melanogaster* (Dm), *A. gambiae* (Am), *A. aegypti* (Aa), *A. mellifera* (Am).

We downloaded amino acid sequences of 124 GSTs from *B. mori* (21), *Drosophila melanogaster* (37), *Anopheles gambiae* (31), *Aedes aegypti* (29), and *Apis mellifera* (6). These and MsGST-msolf1 (NCBI accession number: AAD28279), HaGST-olf (NCBI accession number: AAL23839) and PxGST-cs1 (NCBI accession number: BAD99564) were used to construct the phylogenetic tree (Figure 2). Phylogenetic analysis showed that BmGSTD4 was grouped as a δ class GST. BmGSTD4 and BmGSTD1 form a cluster with MsGST-msolf1 and HaGST-olf. BmGSTD1 was preferentially expressed in the olfactory organs of larvae and moth [19]. Based on previous studies showing that MsGST-msolf1 and HaGST-olf are involved in olfaction [15,16], we hypothesized that the *B. mori* orthologs had similar functions, with BmGSTD4 being involved in silkworm olfaction.

### 2.3. Structural Characterization of BmGSTD4

**Figure 3.**
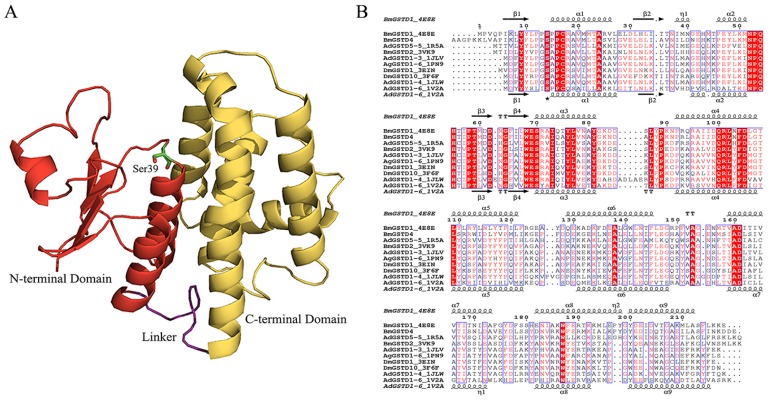
Structural characterization of BmGSTD4. (**A**) Homology model of BmGSTD4. Red, *N*-terminal domain; yellow, *C*-terminal domain; purple, linker. Line, Ser39, which is essential for catalysis; (**B**) Multi-sequence alignment of δ-class GSTs from insects. Amino acid sequences of δ class GSTs with resolved structures are from PDB [27]. PDB ID 4E8E, 1R5A, 3VK9, 1JLV, 1PN9, 3EIN, 3F6F, 1JLW, 1V2A). Asterisk, serine essential for catalysis.

The homology structure of BmGSTD4 was obtained via SWISS-MODEL [20–22] using the known structure of BmGSTD1 (Protein Data Bank (PDB) ID: 4E8E) as a template. A detailed homology model was generated by the website. The sequence conformity between the template and BmGSTD4 was 69.95% with *E*-value 5.27869e^−87^ and QMEAN *Z*-Score [23] −2.309 for the BmGSTD4 model. The root-mean-square deviation (RMSD) between the model and 4E8E was 0.038 Å. The overall structure of the homology model is shown in Figure 3A. The overall structure of BmGSTD4 had a canonical GST fold with two distinct domains connected by a short linker. The *N*-terminal domain consisted of a typical βαβαββα motif topology and the *C*-terminal domain had six helices resembling a right-handed α-helical bundle. Multisequence alignment showed that the *N*-terminal domain of BmGSTD4 with the GSH-binding site (G-site) was conserved, implying a common GSH-binding mechanism. Ser39 is proposed to be catalytically essential [24–26]. However, the *C*-terminal domain with the hydrophobic substrate-binding site (H-site) had relatively low sequence identity with other GSTs. This result was compatible with H-site sequence variations that are responsible for differences and diversities in substrate selectivity.

### 2.4. Antenna-Specific of BmGSTD4

**Figure 4.**
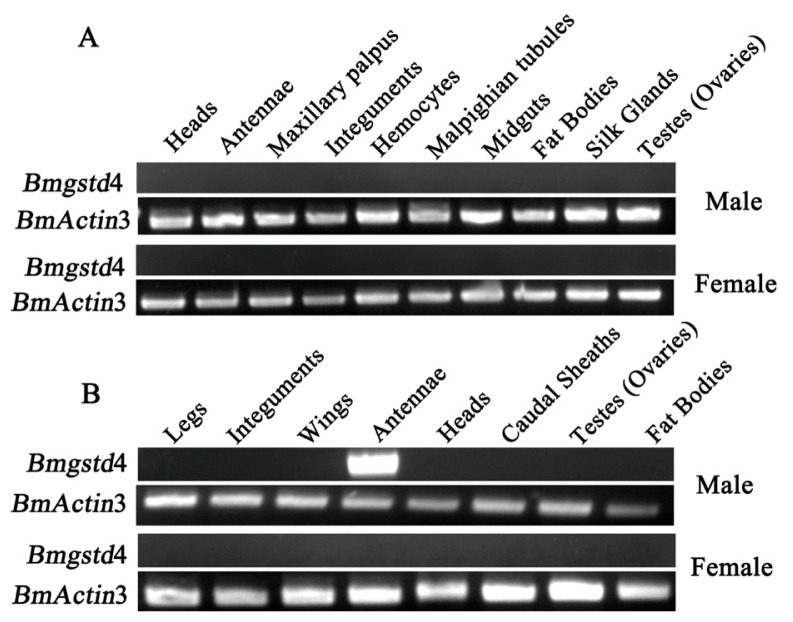
Expression patterns of *Bmgstd*4. (**A**) Expression profiles of *Bmgstd*4 in various tissues from day 3 fifth instar larvae and (**B**) adult moths.

Expression of *Bmgstd*4 in different tissues at larval and adult stages was studied using reverse-transcription polymerase chain reaction (RT-PCR), with no detection in any tissues investigated from day 3 fifth instar larvae (Figure 4A). More thorough examination of expression profiles of larvae, pupae, and adult moths was performed with no detection in larvae or pupae (data not shown). A weak signal was detected in day 1 adult male moths, but not adult female moths. Therefore, we examined the expression of *Bmgstd*4 in various tissues of day 1 adult moths (Figure 4B). The results showed that *Bmgstd*4 was highly and specifically expressed in male silkmoth antennae.

**Figure 5.**
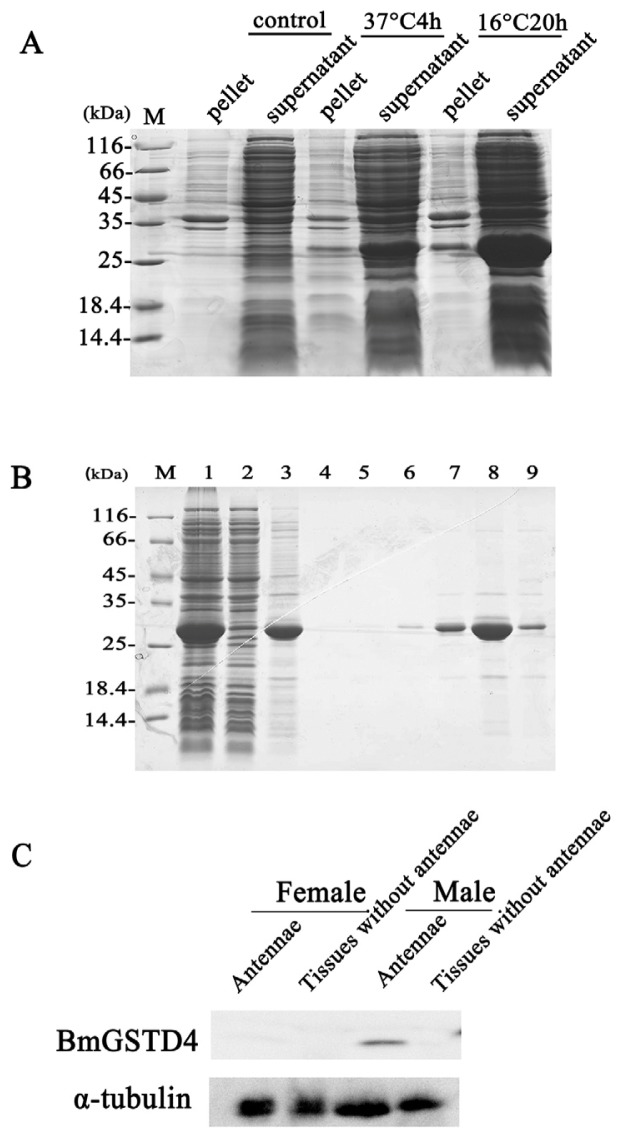
Purification of recombinant BmGSTD4 from *Escherichia. coli* and Western blotting of BmGSTD4. (**A**) Expression of recombinant BmGSTD4 in *E. coli*; (**B**) Purification of recombinant BmGSTD4 using a nickel-chelating column. M: standard protein marker. Lane 1: supernatant from the pretreated sample; Lane 2: flow through; Lane 3–9: sample eluted with binding buffer using a step gradient concentration of imidazole 0, 20, 50, 100, 200, 500, and 1000 mM. Target protein was eluted in binding buffer with 500 mM imidazole; (**C**) Antenna-specificity assessment of BmGSTD4 on Western blot. BmGSTD4 was expressed specifically in silkmoth antennae.

To confirm the antenna specificity of BmGSTD4 at the protein level, recombinant BmGSTD4 protein was overexpressed in *E. coli* and purified (Figure 5A,B). Recombinant BmGSTD4 was expressed in supernatants under growth conditions of 16 °C and induction with isopropyl β-d-1-thiogalactopyranoside (IPTG), and compared to the control. Purified protein (>95% purity) showed a single band on SDS-PAGE of approximately 26 kDa, similar to the calculated molecular weight. Multiple rabbit antibodies for recombinant BmGSTD4 were produced. Western blots showed that BmGSTD4 was expressed only in male silkmoth antennae (Figure 5C).

**Figure 6.**
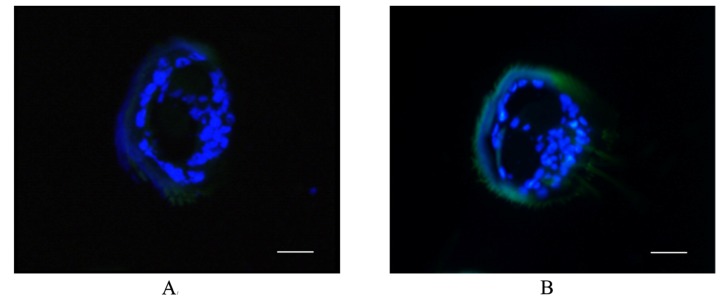
Localization of BmGSTD4. (**A**) Negative control for immunohistochemistry; (**B**) Immunohistochemistry of the BmGSTD4 protein in adult male silkmoth antennae showing distribution in the sensillum of antennae. Bar, 50 μm.

BmGSTD4 localization was investigated via immunohistochemistry. Based on the Western blot results, only adult male silkmoth antennae tissue was investigated for distribution of BmGSTD4. Signals were detected in the sensillum of antennae (Figure 6) and no signal was detected in the negative control. BmGSTD4 had a signal peptide, so the mature polypeptide could be secreted into the sensillum lymph, consistent with the immunohistochemistry results. The results indicated that *Bmgstd*4 was antenna-specific in male silkmoths and might have a particular function in the antennae. In addition to BmGSTD4, we identified BmGSTD1, another δ-class GST in silkworms, which was preferentially expressed in the olfactory organs of larvae and moth [19]. Both represent two novel olfactory-related GSTs in silkworms. Compared with reported olfactory-related GSTs in insects [15,17], BmGSTD4 showed restricted expression in the antennae of male silkmoths.

### 2.5. Enzymatic Properties and GST Activity of BmGSTD4

**Figure 7.**
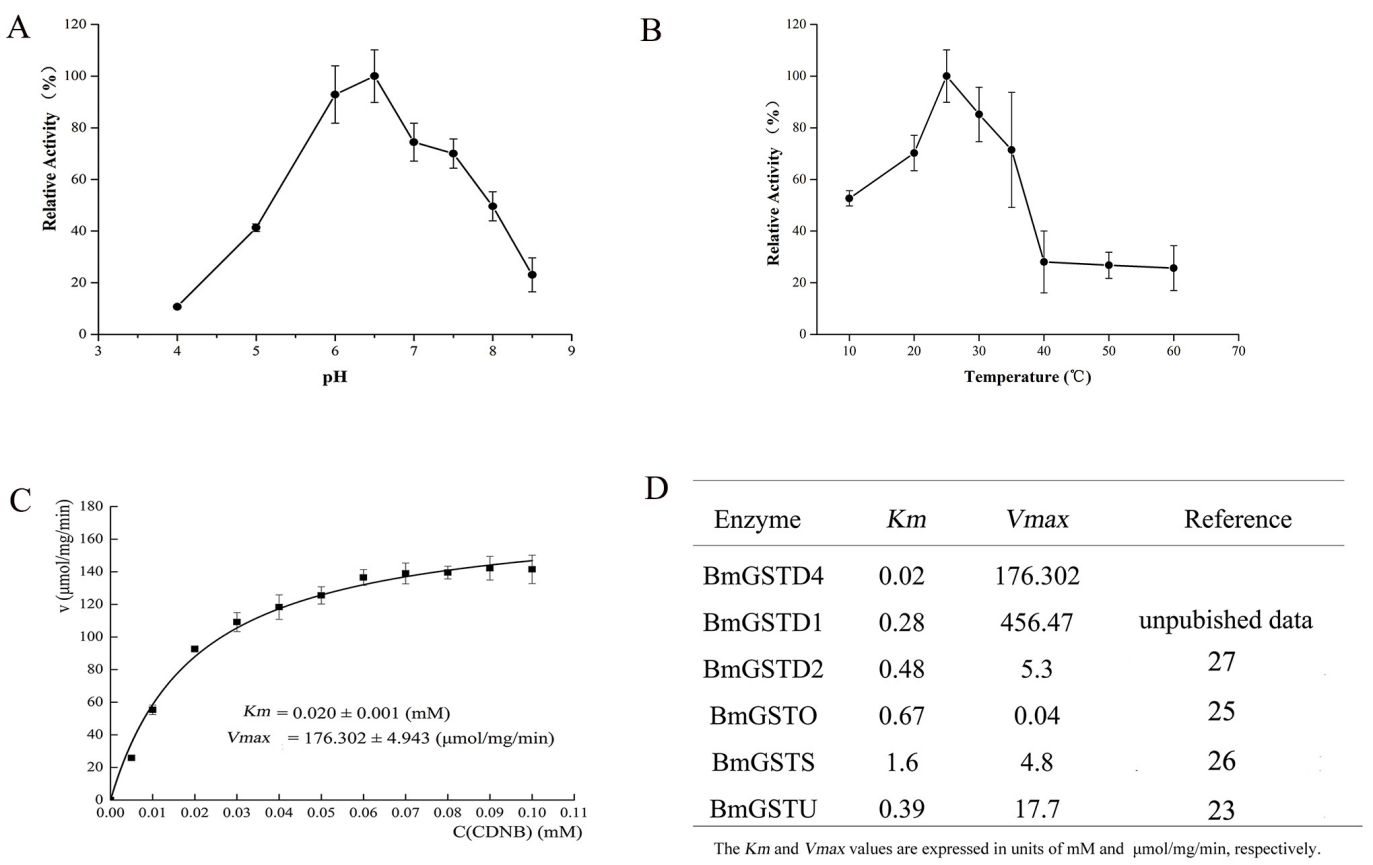
Enzymatic Properties and GST activity of BmGSTD4. (**A**) Optimum pH of recombinant BmGSTD4 assayed at 100 mM acetate-phosphate buffer at various pHs; (**B**) Optimum temperature of recombinant BmGSTD4. Activity was assayed by preincubation of the enzyme solution at various temperatures; (**C**) GST activity of BmGSTD4 with indicated concentrations of CDNB and a fixed concentration of GSH; (**D**) Kinetic data from BmGSTs in the literature.

The enzymatic properties of BmGSTD4 were determined using purified recombinant BmGSTD4 with 1-chloro-2,4-dinitrobenzene (CDNB) and GSH as substrates. The pH optimum of BmGSTD4 was 6 to 6.5 (Figure 7A). BmGSTD4 was stable up to 35 °C (Figure 7B). Above 40 °C, BmGSTD4 retained about 20% of its 25 °C activity.

Recombinant BmGSTD4 showed GSH-conjugating activity towards CDNB. The *Km* was 0.020 mM and *Vmax* was 176.302 μmol/mg/min (Figure 7C). The GST activity of BmGSTD4 showed that it might protect adult male moth antennae against potentially toxic chemicals.

CDNB is insoluble and toxic, and a classic substrate for examining the detoxification activity of GST. Similar to other GSTs, BmGSTD4 had GSH-conjugation activity towards CDNB. From steady-state kinetic analysis using CDNB and GSH we determined *Km* and *Vmax*. In previous studies, several GSTs from *B. mori* were identified and kinetic data were determined with CDNB [28–32] (Figure 7D). BmGSTD4 had the highest GSH-conjugating activity towards CDNB. With specific localization of BmGSTD4 in male silkmoth antennae, these results indicated that BmGSTD4 might be important for detoxification of toxic compounds to protect antennae.

## 3. Discussion

This study identified BmGSTD4, an antenna-specific GST in male adult moths from the silkworm *B. mori*. We cloned the full-length mRNA of *Bmgstd*4 by RACE-PCR. Phylogenetic analysis results showed that BmGSTD4 formed a distinct cluster with MsGST-msolf1 and HaGST-olf, which are olfactory GSTs. Recombinant BmGSTD4 was overexpressed and purified, and showed GST activity towards the classic substrate CDNB. The antenna specificity of *Bmgstd*4 was characterized by transcript and protein expression. BmGSTD4 was localized in the sensillum of antennae. The restricted expression of BmGSTD4 in male silkmoth antennae might represent unique functions.

Homology structures of BmGSTD4 showed similarity to BmGSTD1 (PDB ID: 4E8E) and BmGSTD2 (PDB ID: 3VK9) [33] with RMSD 0.038 Å for BmGSTD1 and 0.749 Å for BMGSTD2. BmGSTD4 is an insect-specific δ-class GST with a canonical GST fold. The conserved *N*-terminal domain suggested that BmGSTD4 had GSH-binding interactions common to known insect δ-class GSTs. Ser39 appeared to be significant for enzyme catalysis. The H-site sequence of BmGSTD4 showed variations responsible for differences in substrate selectivity.

The olfactory system is important for insects to obtain information from the environment. However, olfactory tissues of insects are exposed to xenobiotics and natural compounds, some of which are toxic or affect signal transduction. Therefore, tissue-specific enzymes such as olfactory GSTs are expressed for metabolizing and degrading xenobiotics [15,17,34]. GSTs are multifunctional enzymes involved in the detoxification of hydrophobic and electrophilic toxicants ranging from drugs, herbicides, insecticides to environmental pollutants. The tissue-specific and species-specific expression and distribution of GSTs are considered an adaptive response to the toxicity of endogenous and exogenous metabolites [1,35,36]. Detailed functions of olfactory GSTs need to be clarified and olfactory GSTs are being studied [18]. Olfactory GSTs from insects have been identified [15,17] and various functions in the olfactory system have been proposed. GST-msolf1 in *M. sexta* moths is proposed to protect the olfactory system and inactivate aldehyde odorants. GST-pxcs1 from *P. xuthus* might protect olfactory organs and alternatively or additionally be involved in chemosensory signal transduction. Antennae are important organs for adult silkmoth and are crucial in mating and oviposition sites because they recognize and discriminate between diverse chemicals, such as pheromones. The restricted expression of BmGSTD4 in male silkmoth antennae suggested that BmGSTD4 GST activity was important because BmGSTD4 might detoxify toxic compounds attacking the antennae. BmGSTD4 contained a signal peptide, so it could be secrete into the sensilla lumen and bind hydrophobic ligands, as proposed previously [3,6–8,37]. BmGSTD4 might act as extracellular odorant carrier, and we tested BmGSTD4 binding to odorants. Since BmGSTD4 was antenna specific, functional analysis of BmGSTD4 in addition to detoxification will be studied in the future.

## 4. Experimental Section

### 4.1. Materials and Tissue Collection

The silkworm strain DAZAO, kept in the State Key Laboratory of Silkworm Genome Biology (Southwest University, Chongqing, China), was bred routinely on mulberry leaves at 25 (±1) °C with a relative humidity of 60%–75% until eclosion. Silk glands, testes, ovaries, fat bodies, midguts, integuments, hemocytes, Malpighian tubules, heads, maxillary palpus, and antennae from day 3 fifth instar larvae were dissected on ice. Tissues from male and female moths were dissected into legs, integument, wings, caudal sheaths, gonads, heads, and antennae. All tissues were immediately frozen in liquid nitrogen and stored at −80 °C. For Western blots, only male and female moth antennae were dissected, with other tissues used as controls.

### 4.2. Extraction of RNA and Synthesis of First-Strand cDNA

Total RNA was isolated from dissected tissues using TRIzol reagent (Invitrogen, Carlsbad, CA, USA), and the RNA concentration was determined using a NanoDrop 1000 spectrophotometer (Thermo Scientific, Waltham, MA, USA). First-strand cDNA was synthesized using M-MLV reverse transcriptase (Invitrogen, Carlsbad, CA, USA) following the manufacturer’s instructions. The silkworm cytoplasmic actin *A3* gene (GenBank accession number: NM_001126254) was used as an internal control to normalize cDNAs.

### 4.3. RT-PCR

RT-PCR primers were designed based on reported sequences [38] with forward (F) and reverse (R) primer pairs: F (5′ ATGCTGACAGCGAGTGTCTTGGGAG 3′) and R (5′ TCATTCATCATCCTTAT TTATAAAC 3′). PCR amplification was performed in 25 μL containing normalized cDNA, 15 pmol of each primer, 2 mM MgCl_2_, 0.25 mM dNTP, and 2.5 units Taq DNA polymerase (Takara, Dalian, China) in supplied buffer. PCR was performed at 95 °C for 10 min; 30 cycles of 95 °C for 45 s, 60 °C for 40 s, and 72 °C for 1.5 min, and 72 °C for 10 min. Amplification products were analyzed on 1.5% agarose gels.

### 4.4. Rapid Amplification of cDNA Ends of Bmgstd4

The full-length cDNA of *Bmgstd*4 was cloned using a GeneRacer Kit (Invitrogen, Carlsbad, CA, USA) according to the user manual. Gene-specific primers and nested gene-specific primers for 5′- and 3′-rapid amplification of cDNA ends (RACE) were designed from known sequences [38]: 5′ GSP primer (5′ CTCTGCACCGGTCTGGTCGATGT 3′), 5′ nested GSP primer (5′ GGGTTCTTAGGGTACAACGCATCGTT 3′), 3′ GSP primer (5′ GAATCCTCAACATACCATACCGACT 3′) and 3′ nested primer (5′ ATCGACCAGACCGGTGCAGAGA 3′). RACE products were obtained from 1.5% agarose gels using gel extraction kits (Watson Biotechnologies, Shanghai, China), and purified fragments were cloned into pEasy-T1 simple vector (TransGen, Beijing, China) and sequenced (Sangon, Shanghai, China).

### 4.5. Sequence Analysis

The ORF of *Bmgstd*4 was predicted using analysis tools. The deduced amino acid sequence was aligned with the NCBI Blast website using the PDB database, and analyzed on ExPASy server [39] for biochemical parameters, SignalP 4.1 server [40] for signal peptides, and SMART server [41] for conserved domains.

### 4.6. Phylogenetic Analysis

Amino acid sequences of GSTs from *B. mori* were downloaded from silkDB [42,43], and sequences of *D. melanogaster*, *A. gambiae*, *A. aegypti* and A. *mellifera* were searched in NCBI according to GSTs used in a previous study [44]. Sequences of olfaction GSTs, MsGST-msolf1, HaGST-olf, and PxGST-cs1 were also downloaded. The phylogenetic tree of insect GSTs was reconstructed by Neighbor-Joining (NJ) methods in the program MEGA 6.0 [45]. The evolutionary distance was determined with the Jones-Tayor-Thornton model amino acid matrix. The complete deletion option was used in tree construction and the tree topology accuracy was assessed by 1000 bootstrap replicates.

### 4.7. Homology Modeling of BmGSTD4

The homology structure model of BmGSTD4 was generated by submitting the amino acid sequence without signal peptide to SWISS-MODEL [20–22]. An automated model was created with the resolved structure of BmGSTD1 (PDB ID: 4E8E). Model and quality information were exported in pdf format. Pictures of three-dimensional structures were generated with PyMOL [46]. The amino acid sequences of insect δ-class GSTs with resolved structures were obtained from the PDB database [27]. Multisequence alignments were performed in MultAlign [47] and graphed in ESPript [48].

### 4.8. Expression and Purification of Recombinant BmGSTD4

Recombinant BmGSTD4 without signal peptide was expressed in an *E. coli* system. To obtain soluble recombinant protein, the amino acid sequence was truncated at the first two residues of glycine and proline. Primers for expression were: Forward 5′-GCTA*CATATG*AAAAAGCTAGTGGCGCCTATAAAAC-3′ and Reverse 5′-CCG*CTCGAG*TCATTCATCATCCTTATTTATAAAC-3′. *Nde* I and *Xho* I recognition sites were incorporated into primers (underlined). PCR fragments were purified using PCR cleanup kits (AxyGen, Union, CA, USA) and subcloned into p28 vector (derived from pET28a, Novagen, Darmstadt, Germany). Recombinant clones were validated by restriction digestion and sequenced in both directions. The recombinant plasmid *Bmgstd*4-p28 was transformed into *E. coli* strain Rosetta (DE3) (Novagen, Darmstadt, Germany) competent cells for protein expression. To detect expression, *E. coli* competent cells without transformation were used as a negative control. Protein-expressing cells were grown at 37 °C using 2× YT culture medium (5 g NaCl, 16 g bacto-tryptone, 10 g yeast extract/L) until approximately 0.6–0.8 optical density (OD) at 600 nm. Isopropyl IPTG was added to a final concentration of 0.1 mM. Cells were harvested after 20 h at 16 °C and homogenized in lysis buffer (100 mM NaCl, 20 mM Tris, pH 8.5), and sonicated in ice water. After centrifugation at 16,000 rpm at 4 °C for 30 min, target proteins in supernatants were purified by a HiTrap nickel-chelating column (Qiagen, Venlo, Netherlands) and a HiLoad 16/60 desalting column (GE Healthcare, Little Chalfont, UK) equilibrated with 100 mM NaCl, 20 mM Tris, pH 8.0. Protein purity was assayed by SDS-PAGE. Purified recombinant protein was concentrated by ultrafiltration (Millipore Amicon, Billerica, MA, USA) and stored at −20 °C in 1 mM DTT and 40% glycerol.

### 4.9. Western Blotting and Immunochemistry

Antibodies for recombinant BmGSTD4 were produced by immunizing a rabbit with purified recombinant protein. Total proteins were extracted from antennae and other tissues of male and female silkmoths after grinding in liquid nitrogen and lysis in RIPA lysis buffer (Beyotime, Shanghai, China). The concentration of extracted protein was determined using Bradford Protein Assay kits (Beyotime, Shanghai, China). Samples containing equivalent proteins (80 μg) were separated via SDS-PAGE (12.5% (*w*/*v*) polyacrylamide gel) and transferred to PVDF membranes (Roche, Branford, CT, USA). Membranes were blocked with 1% (*w*/*v*) dry skim milk overnight and incubated with antibody at 1:5000 with Tris-buffered saline containing Tween 20 (TBST) and washed with TBST. Horseradish peroxidase-labeled goat anti-rabbit IgG (H+L) (Sigma-aldrich, St. Louis, MO, USA) as secondary antibody was added at 1:10,000 with TBST, followed by washing with TBST. Signals were detected with Amersham ECL Advance Western Blotting Detection Kits (GE Healthcare, Little Chalfont, UK) using an imaging system (CLiNX, Shanghai, China).

Antennae sections were dissected from day 1 adult male silkmoths. Sections were fixed in 4% paraformaldehyde at 4 °C overnight, embedded in paraffin and cut to 5–6 μm. Sections were blocked with goat serum at 37 °C for 1 h. Primary antibodies were used at a dilution of 1:100 and the FITC-labeled goat anti-rabbit IgH (H+L) (Sigma) was used as a secondary antibody at 1:300. DAPI displays blue fluorescence under UV light. Sections were examined under a BX51 inverted fluorescence microscope (Olympus, Tokyo, Japan).

### 4.10. Enzymatic Assays

Enzymatic activity assays for BmGSTD4 used CDNB and GSH as standard substrates and a DU800 spectrophotometer (Beckman Coulter, Brea, CA, USA). Optimal pH and temperature measurements used fixed concentrations of CDNB (0.25 mM) and GSH (1 mM). Optimum pH for activity was assayed using acetate-phosphate buffer at pH 4.0, 4.5, 5, 6.0, 6.5, 7.0, 7.5, 8.0 or 8.5. Thermostability was determined by preincubation of the enzyme solution at 10, 20, 25, 30, 35, 40, 50, or 60 °C before activity was assayed. GST activity assay conditions with different concentrations of CDNB and 1 mM GSH were in 0.1 M phosphate buffer (pH 6.5) under conditions described previously [49]. Reactions were monitored at 340 nm, ɛ = 9600 M^−1^·cm^−1^.

## 5. Conclusions

In summary, we identified BmGSTD4, an antenna-specific GST in male silkmoths. The full-length mRNA of *Bmgst*d4 was successfully cloned. BmGSTD4 was a typical GST belonging to the δ class and adopted a canonical GST fold. Recombinant BmGSTD4 showed GST activity towards the reference substrate CDNB. Antenna specificity of *Bmgstd*4 in male silkmoths was characterized at the mRNA and protein levels. BmGSTD4 was localized in the sensillum of antennae. This work presented initial research on BmGSTD4. Future studies will clarify the functions of BmGSTD4 in addition to detoxification.

## References

[1] Hayes J.D., Flanagan J.U., Jowsey I.R. (2005). Glutathione transferases. Annu. Rev. Pharmacol. Toxicol.

[2] Armstrong R.N. (1997). Structure, catalytic mechanism, and evolution of the glutathione transferases. Chem. Res. Toxicol.

[3] Listowsky I., Abramovitz M., Homma H., Niitsu Y. (1988). Intracellular binding and transport of hormones and xenobiotics by glutathione-*S*-transferases. Drug Metab. Rev.

[4] Zhao T., Singhal S.S., Piper J.T., Cheng J., Pandya U., Clark-Wronski J., Awasthi S., Awasthi Y.C. (1999). The role of human glutathione *S*-transferases hGSTA1–1 and hGSTA2–2 in protection against oxidative stress. Arch. Biochem. Biophys.

[5] Johansson A.S., Mannervik B. (2001). Human glutathione transferase A3–3, a highly efficient catalyst of double-bond isomerization in the biosynthetic pathway of steroid hormones. J. Biol. Chem.

[6] Lo Bello M., Nuccetelli M., Caccuri A.M., Stella L., Parker M.W., Rossjohn J., McKinstry W.J., Mozzi A.F., Federici G., Polizio F. (2001). Human glutathione transferase P1–1 and nitric oxide carriers; A new role for an old enzyme. J. Biol. Chem.

[7] Dulhunty A., Gage P., Curtis S., Chelvanayagam G., Board P. (2001). The glutathione transferase structural family includes a nuclear chloride channel and a ryanodine receptor calcium release channel modulator. J. Biol. Chem.

[8] Bhargava M.M., Listowsky I., Arias I.M. (1978). Ligandin. Bilirubin binding and glutathione-*S*-transferase activity are independent processes. J. Biol. Chem.

[9] Mannervik B., Awasthi Y.C., Board P.G., Hayes J.D., di Ilio C., Ketterer B., Listowsky I., Morgenstern R., Muramatsu M., Pearson W.R. (1992). Nomenclature for human glutathione transferases. Biochem. J.

[10] Chelvanayagam G., Parker M.W., Board P.G. (2001). Fly fishing for GSTs: A unified nomenclature for mammalian and insect glutathione transferases. Chem. Biol. Interact.

[11] Enayati A.A., Ranson H., Hemingway J. (2005). Insect glutathione transferases and insecticide resistance. Insect Mol. Biol.

[12] Aceto A., Sacchetta P., Dragani B., Bucciarelli T., Angelucci S., Longo V., Gervasi G.P., Martini F., di Ilio C. (1993). Glutathione transferase isoenzymes in olfactory and respiratory epithelium of cattle. Biochem. Pharmacol.

[13] Banger K.K., Lock E.A., Reed C.J. (1993). The characterization of glutathione *S*-transferases from rat olfactory epithelium. Biochem. J.

[14] Ben-Arie N., Khen M., Lancet D. (1993). Glutathione *S*-transferases in rat olfactory epithelium: Purification, molecular properties and odorant biotransformation. Biochem. J.

[15] Rogers M.E., Jani M.K., Vogt R.G. (1999). An olfactory-specific glutathione-*S*-transferase in the sphinx moth *Manduca sexta*. J. Exp. Biol.

[16] Wang G.-R., Guo Y.-Y., Wu K.-M. (2004). Cloning of a cDNA fragment of an antenna-specific gene in *Helicoverpa armigera*. Chin. J. Agric. Biotechnol.

[17] Ono H., Ozaki K., Yoshikawa H. (2005). Identification of cytochrome P450 and glutathione-*S*-transferase genes preferentially expressed in chemosensory organs of the swallowtail butterfly, *Papilio xuthus* L. Insect Biochem. Mol. Biol.

[18] Espinoza H.M., Shireman L.M., McClain V., Atkins W., Gallagher E.P. (2013). Cloning, expression and analysis of the olfactory glutathione *S*-transferases in coho salmon. Biochem. Pharmacol.

[19] Tan X., Guo P., Hu X., Chen Q., Zhao P., Xia Q., Zhou C., Southwest University, Chongqing, China (2014). Structural and biochemical characterisation of *Bombyx mori* glutathione *S*-transferase BmGSTD1 preferentially expressed in the olfactory organs.

[20] Kiefer F., Arnold K., Kunzli M., Bordoli L., Schwede T. (2009). The SWISS-MODEL repository and associated resources. Nucleic Acids Res.

[21] Arnold K., Bordoli L., Kopp J., Schwede T. (2006). The SWISS-MODEL workspace: A web-based environment for protein structure homology modelling. Bioinformatics.

[22] Schwede T., Kopp J., Guex N., Peitsch M.C. (2003). SWISS-MODEL: An automated protein homology-modeling server. Nucleic Acids Res.

[23] Benkert P., Biasini M., Schwede T. (2011). Toward the estimation of the absolute quality of individual protein structure models. Bioinformatics.

[24] Allocati N., Federici L., Masulli M., di Ilio C. (2009). Glutathione transferases in bacteria. FEBS J.

[25] Sheehan D., Meade G., Foley V.M., Dowd C.A. (2001). Structure, function and evolution of glutathione transferases: Implications for classification of non-mammalian members of an ancient enzyme superfamily. Biochem. J.

[26] Armstrong R.N. (2000). Mechanistic diversity in a metalloenzyme superfamily. Biochemistry.

[27] Bernstein F.C., Koetzle T.F., Williams G.J., Meyer E.F., Brice M.D., Rodgers J.R., Kennard O., Shimanouchi T., Tasumi M. (1977). The protein data bank: A computer-based archival file for macromolecular structures. J. Mol. Biol.

[28] Yamamoto K., Ichinose H., Aso Y., Banno Y., Kimura M., Nakashima T. (2011). Molecular characterization of an insecticide-induced novel glutathione transferase in silkworm. Biochim. Biophys. Acta.

[29] Yamamoto K., Shigeoka Y., Aso Y., Banno Y., Kimura M., Nakashima T. (2009). Molecular and biochemical characterization of a Zeta-class glutathione *S*-transferase of the silkmoth. Pestic. Biochem. Phys.

[30] Yamamoto K., Nagaoka S., Banno Y., Aso Y. (2009). Biochemical properties of an omega-class glutathione *S*-transferase of the silkmoth *Bombyx mori*. Comp. Biochem. Physiol. C Toxicol. Pharmacol.

[31] Yamamoto K., Zhang P.B., Banno Y., Fujii H. (2006). Identification of a sigma-class glutathione-*S*-transferase from the silkworm *Bombyx mori*. J. Appl. Entomol.

[32] Yamamoto K., Zhang P., Miake F., Kashige N., Aso Y., Banno Y., Fujii H. (2005). Cloning, expression and characterization of theta-class glutathione *S*-transferase from the silkworm *Bombyx mori*. Comp. Biochem. Physiol. B Biochem. Mol. Biol.

[33] Yamamoto K., Usuda K., Kakuta Y., Kimura M., Higashiura A., Nakagawa A., Aso Y., Suzuki M. (2012). Structural basis for catalytic activity of a silkworm Delta-class glutathione transferase. Biochim. Biophys. Acta.

[34] Rybczynski R., Vogt R.G., Lerner M.R. (1990). Antennal-specific pheromone-degrading aldehyde oxidases from the moths *Antheraea polyphemus* and *Bombyx mori*. J. Biol. Chem.

[35] Raza H. (2011). Dual localization of glutathione *S*-transferase in the cytosol and mitochondria: Implications in oxidative stress, toxicity and disease. FEBS J.

[36] Zimniak P., Singh S.P. (2006). Families of glutathione transferases. Toxicology of Glutathione Transferases.

[37] Litwack G., Ketterer B., Arias I.M. (1971). Ligandin: A hepatic protein which binds steroids, bilirubin, carcinogens and a number of exogenous organic anions. Nature.

[38] Yu Q., Lu C., Li B., Fang S., Zuo W., Dai F., Zhang Z., Xiang Z. (2008). Identification, genomic organization and expression pattern of glutathione *S*-transferase in the silkworm *Bombyx mori*. Insect Biochem. Mol. Biol.

[39] Gasteiger E., Gattiker A., Hoogland C., Ivanyi I., Appel R.D., Bairoch A. (2003). ExPASy: The proteomics server for in-depth protein knowledge and analysis. Nucleic Acids Res.

[40] Petersen T.N., Brunak S., von Heijne G., Nielsen H. (2011). SignalP 4.0: Discriminating signal peptides from transmembrane regions. Nat. Methods.

[41] Letunic I., Doerks T., Bork P. (2012). SMART 7: Recent updates to the protein domain annotation resource. Nucleic Acids Res.

[42] Xia Q., Zhou Z., Lu C., Cheng D., Dai F., Li B., Zhao P., Zha X., Cheng T., Chai C. (2004). A draft sequence for the genome of the domesticated silkworm (*Bombyx mori*). Science.

[43] Duan J., Li R., Cheng D., Fan W., Zha X., Cheng T., Wu Y., Wang J., Mita K., Xiang Z. (2010). SilkDB v2.0: A platform for silkworm (*Bombyx mori*) genome biology. Nucleic Acids Res.

[44] Friedman R. (2011). Genomic organization of the glutathione *S*-transferase family in insects. Mol. Phylogenet Evol.

[45] Tamura K., Stecher G., Peterson D., Filipski A., Kumar S. (2013). MEGA6: Molecular evolutionary genetics analysis version 6.0. Mol. Biol. Evol.

[46] DeLano W.L. (2002). The PyMOL Molecular Graphic System.

[47] Corpet F. (1988). Multiple sequence alignment with hierarchical clustering. Nucleic Acids Res.

[48] Gouet P., Courcelle E., Stuart D.I., Metoz F. (1999). ESPript: Analysis of multiple sequence alignments in PostScript. Bioinformatics.

[49] Habig W.H., Pabst M.J., Jakoby W.B. (1974). Glutathione *S*-transferases. The first enzymatic step in mercapturic acid formation. J. Biol. Chem.

